# 

**Published:** 1886-02-20

**Authors:** 


### TABLE OF CONTENTS.

### ORIGINAL AND SELECTED ARTICLES.

Coughs, Remarks made at a Clinic by R. C. Word, M. D , Professor of Physiology in the Southern Medical College, Atlanta, Ga...........41
Suggestions from Dispensary Experience, for the Surgery of General Practice, Read before the Philadelphia County Medical Society, December 10 1884, by Charles W. Dulles, M. D.44
On the Use of Salix Nigra (Aments), a New Sexual Sedative, in the Treatment of Masturbation, Excessive Venery Spermatorrhœa, and Ovarian Disease, by F T. Paine, M. D., Comanche, Texas.........51
Spray Method—what it can do for Catarrh and its Effects, by Hiram Christopher, A M., M.D., Prof. of Chemistry in the St Joseph Medical College, St. Joseph, Mo..........54
Peraldehyde..........56

### ABSTRACTS AND GLEANINGS.

Case of Dystocia..........57
The Treatment of Pleurisy in the Bellevue Hospital..........60
Supra-Pubic Aspiration for Retention of Urine; A New Method for the Removal of Foreign Bodies from the Nose..........61
Diphtheria Cured by Tolu Varnish..........62
Fatal Dose of Sulphate of Quinine; Alkaloid Pomegranates; Cocaine in Gonorrhœa..........63
Cessation of Menstruation; A Parturiometer...64
Forcible Injections in Intestinal Obstruction.....65
A Mode of Treating Acute Inflammation of the Kee-Joint; "A Genuine Case of Hydrophobia Cured."..........66
The Therapeutic Action of Theine; Pichi; Amaurosis Due to Bromide of Potassium..........67
Symptoms Resulting from Swallowing a Cater pillar; Pulverized Vaccine Virus; Jaundice.. 68
Crotonol; Choera; Cocaine in Inflamed Nipples.69
Action of Pilocarpine and Atropine; Premature Labor by Electricity; Reduction of Dislocations by Pressure; Hamamelis in Prostatic Enlargement; Painless Tooth Extraction........70

### SCIENTIFIC ITEMS.

To Glue Leather to Iron; Water is Fattening; Insusceptibility of a Dog to the Action of Hydrocyanic Acid..........71
The Moon and Us; Naturalists..........72

### PRACTICAL NOTES AND FORMULÆ.

Dr. Louries' Cholera Mixture; Squibb's Cholera Mixture; Ruschenberger's Cholera Mixture; Digestive Wine; Good Mucilage; Chronic Nasal Catarrh..........73
Prescription for Apthous Sore Throat; For Constipation in Young Children; To Disguise the Taste of Quinine; Chronic Rheumatism; For Painful Hemorrhage; Anodyne for Children; Chronic Diarrhœa..........74

### EDITORIALS AND MISCELLANEOUS.

Editorial Notices..........75
Atlanta as a Medical Center..........76
Death of Dr. Joseph A. Eve; Commission for Investigating Yellow Fever Inoculation..........77
Pamphlets Received..........79
Receipted; Special Notices..........80

